# Supplementary material for: The TRPV4 channel links calcium influx to DDX3X activity and viral infectivity
Source: Nat Commun. 2018 Jun 13;9:2307. doi: 10.1038/s41467-018-04776-7 (PMC5998047; doi:10.1038/s41467-018-04776-7)
Supplement: Supplementary file 1 — Supplementary Information [file 41467_2018_4776_MOESM1_ESM.pdf]

Supplementary Information

The TRPV4 channel links calcium influx to DDX3X activity and viral infectivity

Doñate-Macian *et al.*

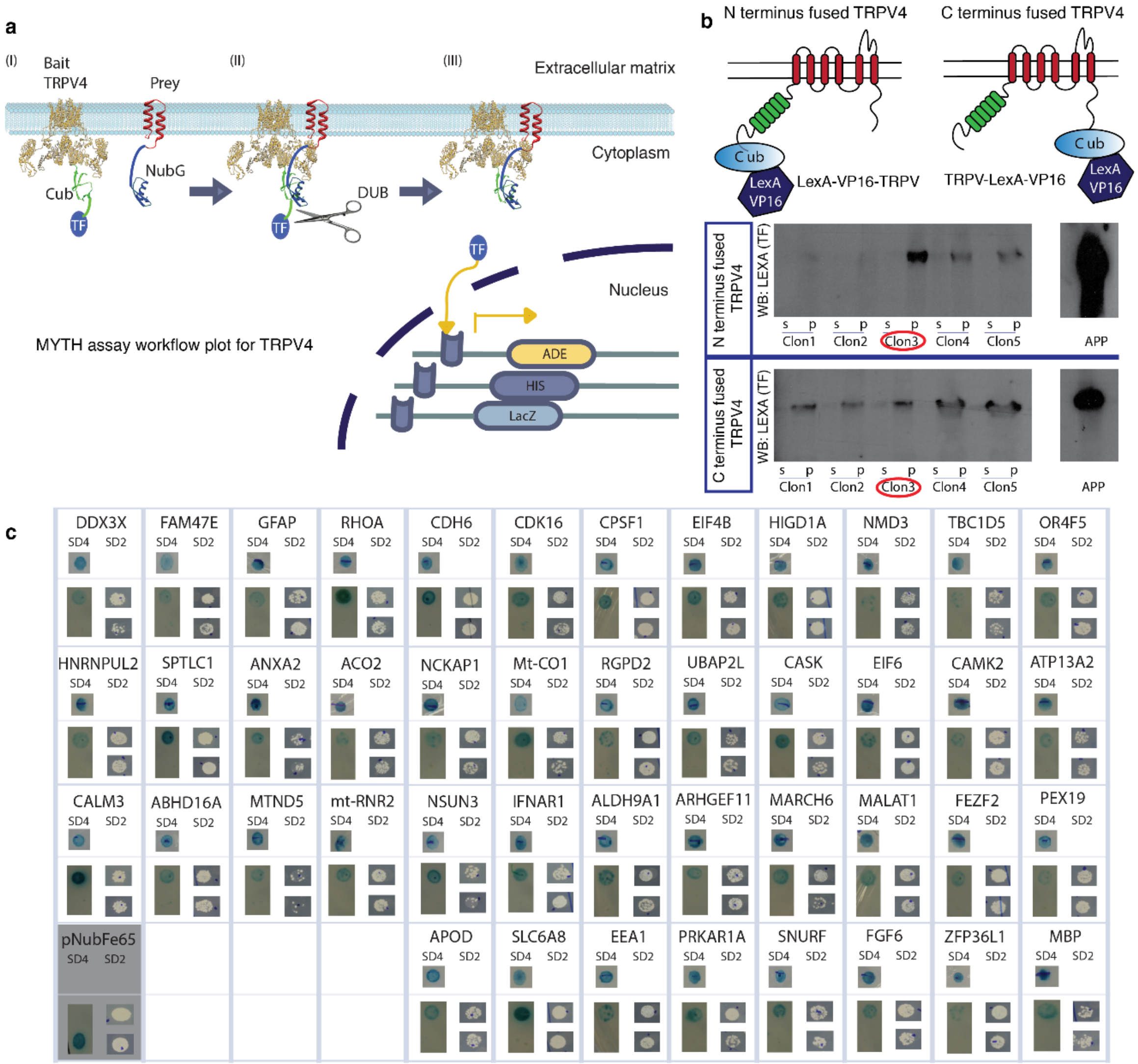

Supplementary Figure 1. TRPV4 Membrane Yeast Two-Hybrid Screen. (**a**) Workflow for MYTH assay. I. TRPV4 tagged with the bait C-terminus of ubiquitin (Cub) and VP16-LexA transcription factor (TF) was transformed in the Yeast reporter strain. II. cDNA library transformation of preys tagged with N-terminus of ubiquitin (NubG). Upon interaction of bait and prey, ubiquitin is refolded and the degradation ubiquitin complex (DUB) releases the TF. III. TF goes to the nucleus and starts the expression of several reporter genes integrated in the strain that codify for auxotrophic markers Histidine, Adenine and ß-galactosidase enzyme. (**b**) Validation of bait expression. TRPV4 N-terminus and C-terminus bait expression confirmed by Western Blot (WB) against LexA. APP fused to bait tag was used as positive control for the WB. (**c**) Positive interaction partners determined by our MYTH screen. Interaction strength can be estimated by the colony blue color intensity due to the B-galactosidase reporter. SD2 (SD-LEU-TRP, non selective media), SD4 (SD-LEU-TRP-HIS containing 7,5mM 3AT and Xgal, selective media). First row within each interactor box corresponds to the colony when firstly isolated from SD4. The second row corresponds to the re-transformation of the TRPV4 bait strain with the bait to confirm the interaction. The third row contains the transformation of the bait within the control strain pNUBFe65, as negative control for the specificity of the interaction.

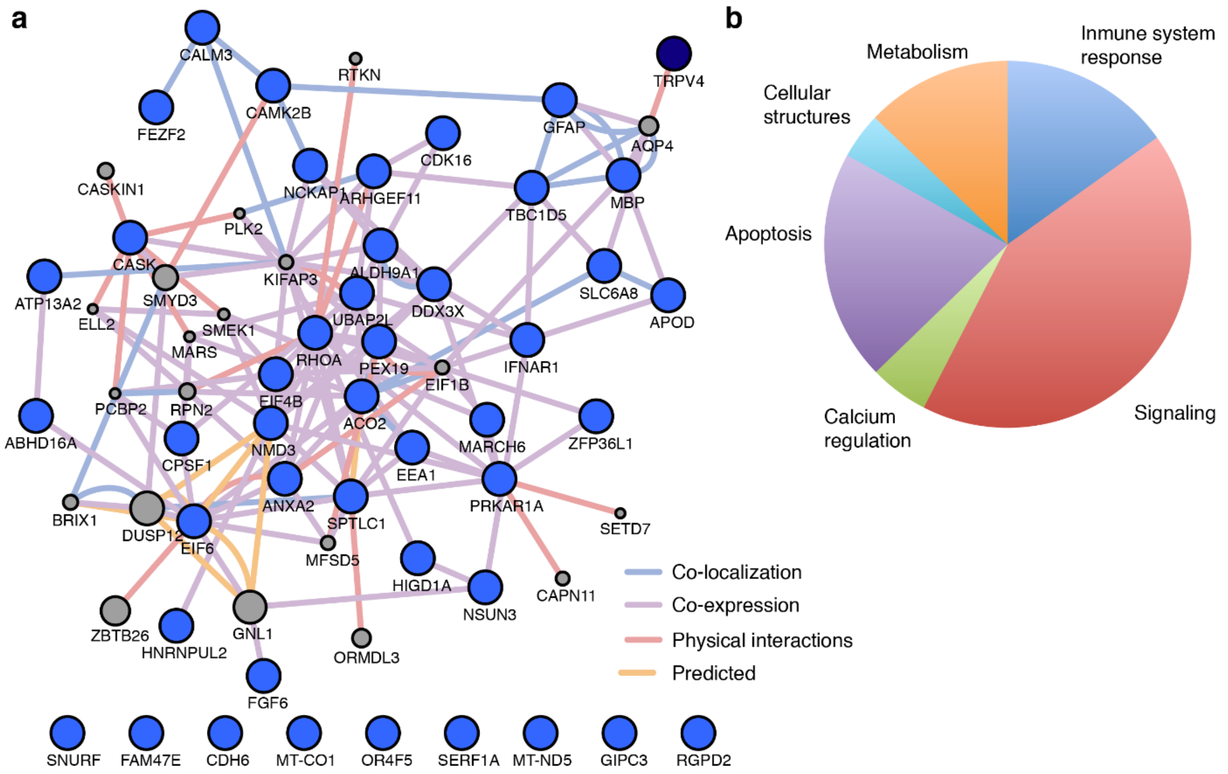

c

Process		Genes	pValue (highest association)
Inmune system response		DDX3, IFNAR1, RHOA	2.4 10-3
Signaling		TRPV4, FGF6, ARHGEF11, PRKA1A, ALD9A1, CamK2B	9.3 10-3
Calcium regulatory process		TRPV4, CALM3, CamK2B	2.2 10-2
Apoptosis		RHOA, ARHGEF11	4.2 10-2
Cellular structures		RHOA, ANXA2	4.2 10-2
Metabolic process		CDK16, MT-CO1, CASK, CamK2B	4.3 10-2

Supplementary Figure 2. Gene enrichment analysis of TRPV4 MYTH screen. (**a**) TRPV4 MYTH enriched interaction network obtained by Genemania algorithm based on protein co-localization, gene co-expression, genetic interactions and domain conservation. Blue dots represent the potential interactors identified by MYTH screen. (**b**) Abundance of statistically significant GO-terms associated to TRPV4 interactome clustered by their function (cellular process). (**c**) MYTH TRPV4 partners and highest p-value associated to each cellular process.

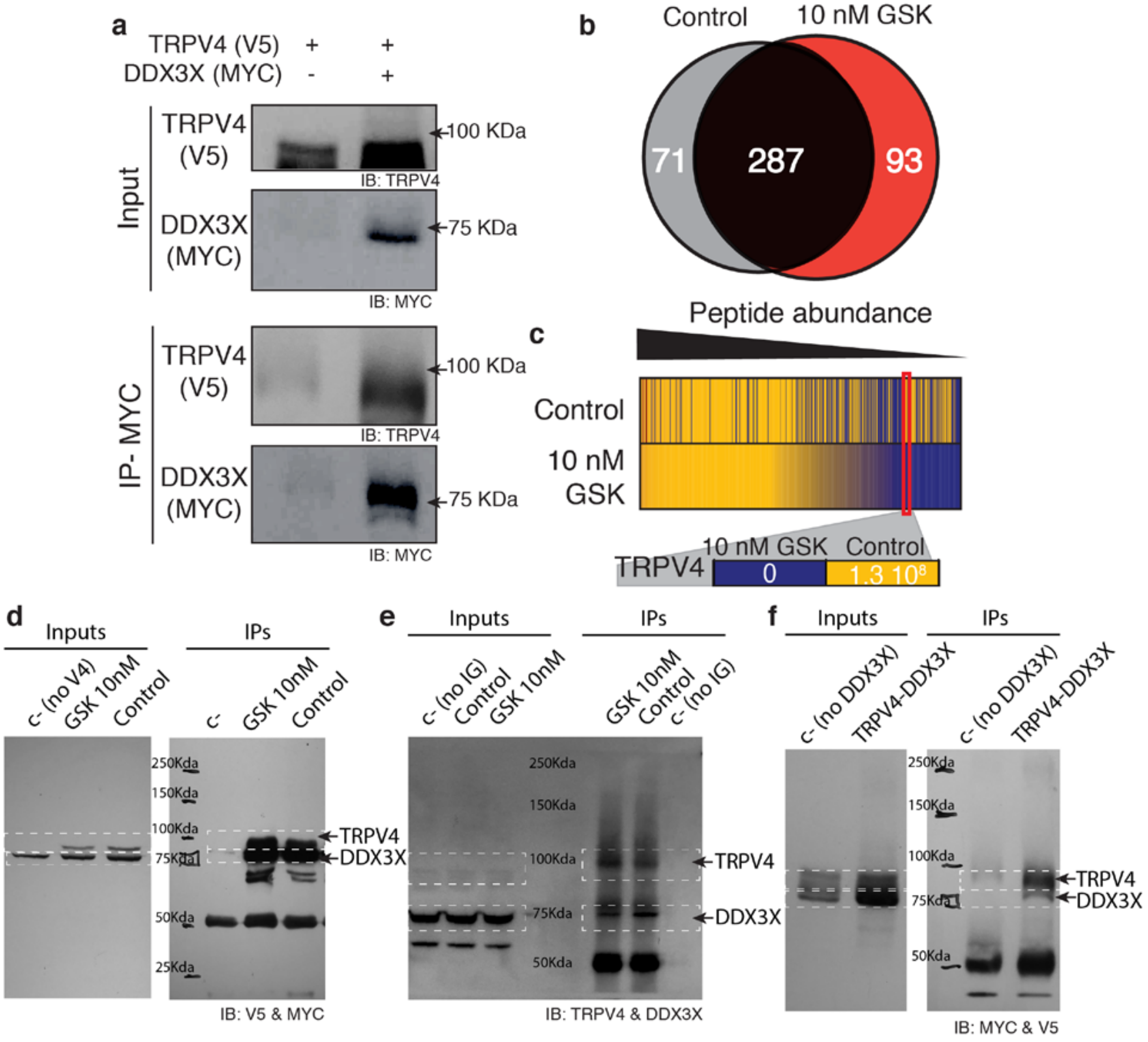

Supplementary Figure 3. TRPV4 interacts with DDX3X. (a) Co-immunoprecipitation of TRPV4-V5(tag) and DDX3X-Myc(tag) in HEK293 cells. (**b**) Mass spectrometry analysis of DDX3X-pull down showing the number of interaction partners identified by MALDI-TOFF in isotonic control media (light grey), in the presence of TRPV4 agonist GSK (dark red) or in both condition (black). (**c**) Heat map of mass spectrometry DDX3X partners arranged according to peptide abundance. The P value is represented as a color scale with yellow being the highest and purple the lowest values. (**d-f**) Uncropped scans of the western blots presented in figures 1a, 1b and supplementary figure 3a.White dotted lines delimitate the areas magnified on the respective figures.

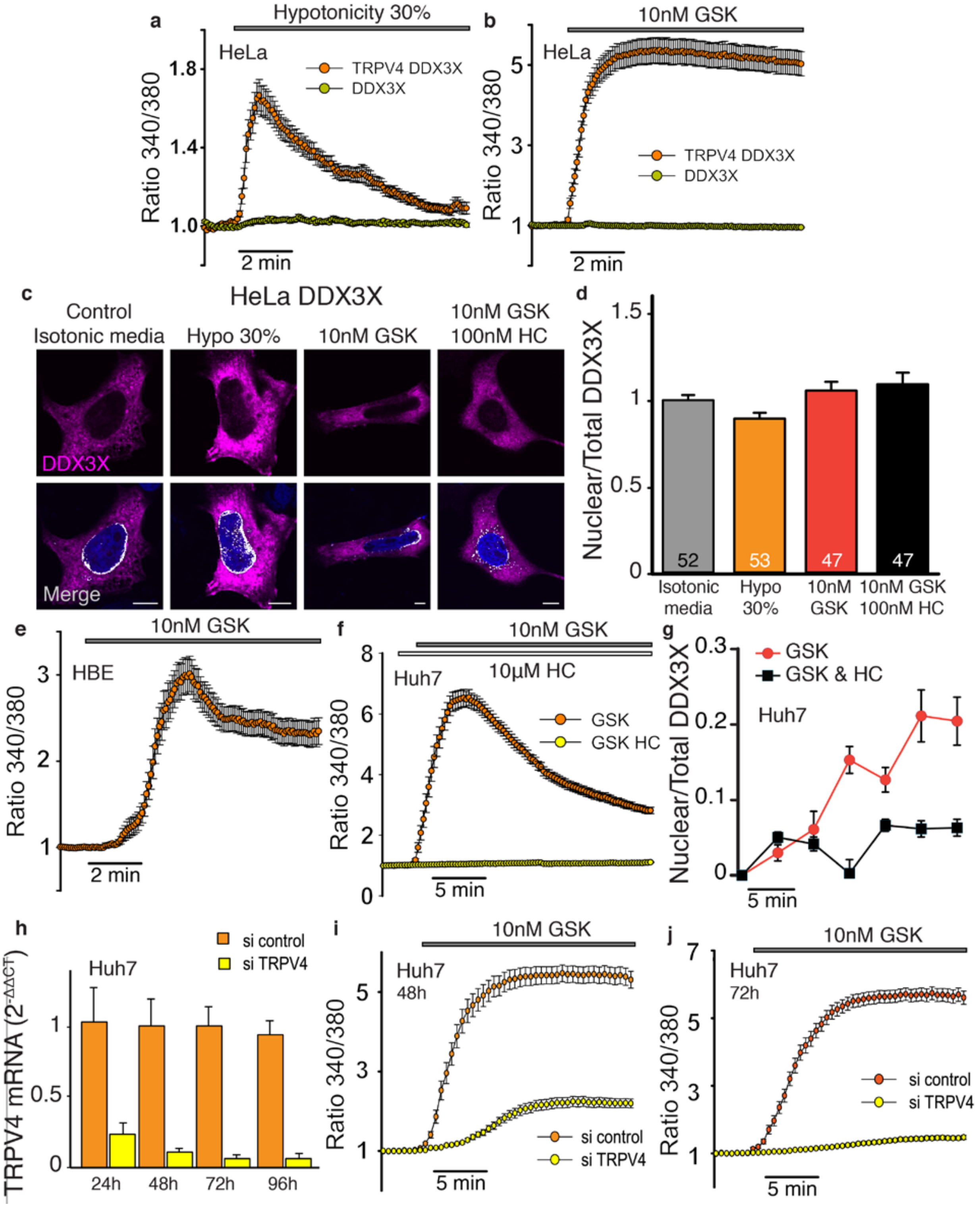

Supplementary Figure 4. DDX3X nuclear shuttling upon TRPV4 activation only happens in cells expressing functional TRPV4 channels. Changes in cytosolic calcium concentration measured as the Fura-2 ratio (340/380) in HeLa cells overexpressing TRPV4 and/or DDX3X-Myc and exposed to 30% hypotonic solutions (**a**) or 10 nM GSK1016790A (**b**). Data are presented as Mean (±SEM) of three

independent experiments with n=94 (TRPV4+DDX3X, hypotonicity), n=96 (TRPV4+DDX3X, GSK1016790A), n=105 (DDX3X, hypotonicity) and n=94 (DDX3X, GSK1016790A). (**c**) Confocal immunofluorescence images of DDX3X (magenta) in HeLa cells overexpressing DDX3X-Myc. Nuclei were stained with DAPI (blue). Colocalization of DDX3X and nuclei is shown in white in the merge panels. Scale bar: 10 μm. (**d**) Mean nuclear/total DDX3X ratio obtained under the experimental conditions indicated in HeLa cells overexpressing DDX3X-Myc. The number of cells analyzed for each condition is indicated in each bar. (**e-f**) Changes in cytosolic calcium concentration measured as the Fura-2 ratio (340/380) in HBE cells and in Huh7 cells in response to 10 nM GSK1016790A with or without 1μM HC067047. (**g**) Nuclear/total DDX3X ratio at different time points after addition of GSK1016790A or GSK1016790A plus HC067047 to Huh7 cells. (**h**) RT-qPCR comparing TRPV4 mRNA levels in Huh7 cells transfected with siControl and siTRPV4. Mean (±SEM) of n=3 experiements. P=0.03, 0.008, 0.002 and 0.001 when comparing siControl with siTRPV4 at the different time points, as determined by two-tailed Student's t-test). Mean (±SEM) of normalized intracellular Ca^{2+} signals activated by GSK1016790A (10 nM) in Huh7 cells 48 h (**i**) and 72 h (**j**) after transfection with siControl (3 independent experiments with n=78 for 48h and n=102 for 72h) and siTRPV4 (3 independent experiments with n=105 for 48h and n=105 for 72h).

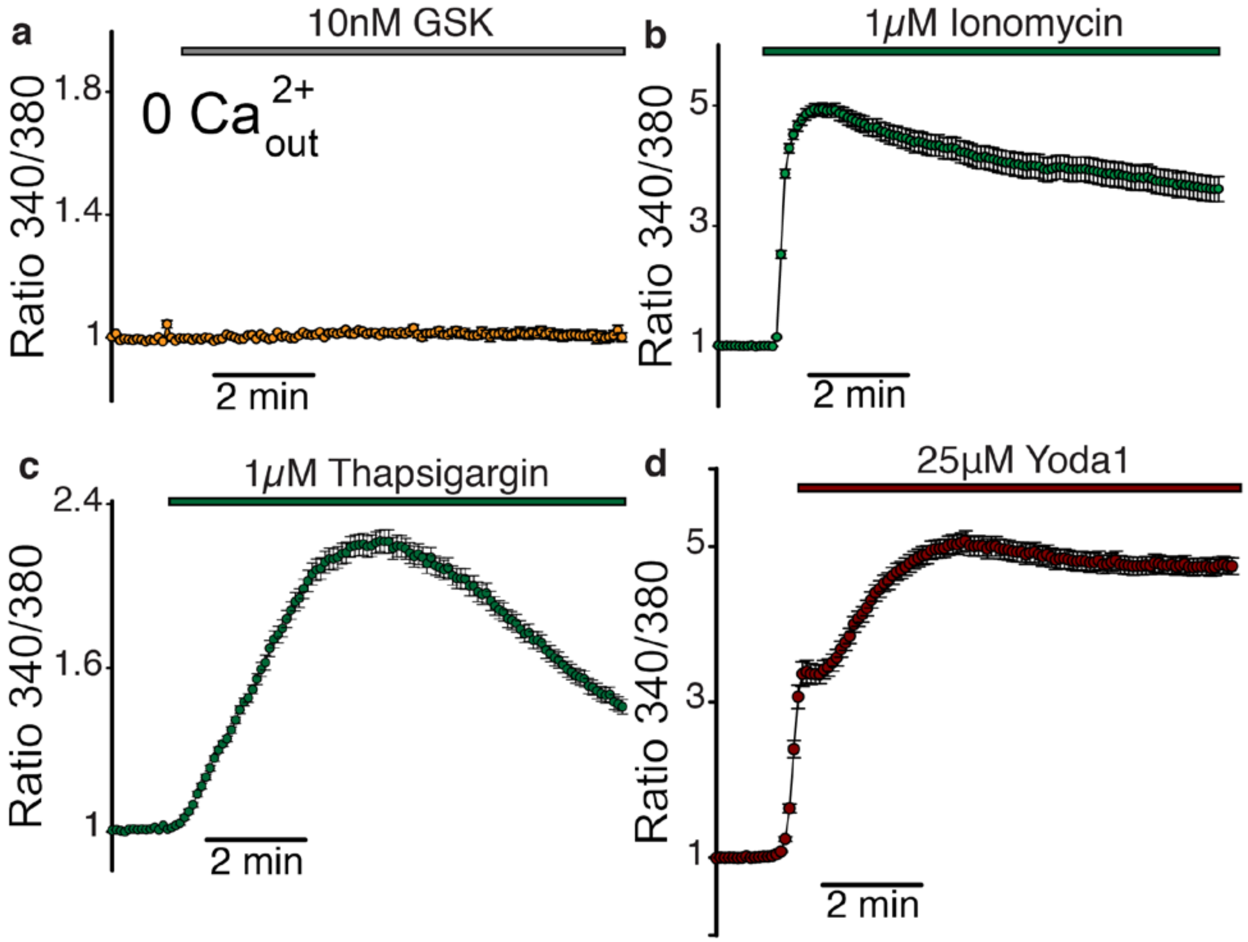

Supplementary Figure 5. **Changes in intracellular calcium concentration in HeLa cells transiently transfected with TRPV4 and DDX3X.** Changes in cytosolic calcium concentration measured as the Fura-2 ratio (340/380) in HeLa cells overexpressing TRPV4 and DDX3X-Myc and exposed to 10 nM GSK1016790A in the absence of extracellular Ca^{2+} (**a**), 1 μM ionomycin (**b**), 1 μM thapsigargin (**b**) and 25 μM Yoda 1 (**d**). Data are presented as Mean (±SEM) of six independent experiments with n=104 (a), n=100 (b), n=103 (c) and n=104 (d).

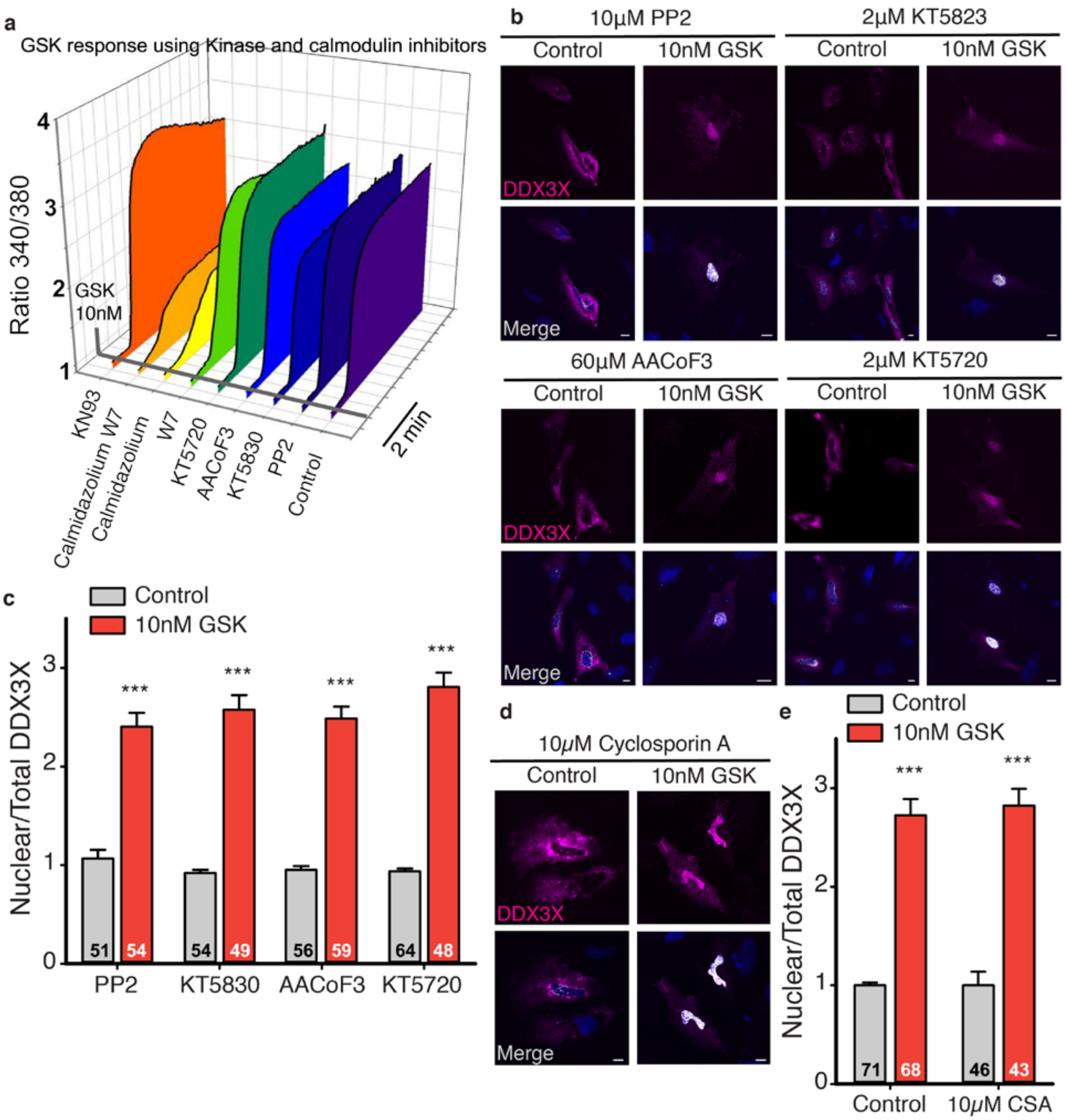

Supplementary Figure 6. TRPV4 downstream signaling. TRPV4-dependent DDX3X nuclear shuttling is not mediated by SRCs, PKG, PLA2, PKA or Calcineurin. (a) Changes in intracellular Ca^{2+} concentration (indicated by normalized fura-2 ratios) in HeLa cells transfected with TRPV4 and DDX3X and treated with GSK1016790A in the absence (Control) or presence of the indicated drugs. Data are presented as Mean (±SEM) of six independent experiments with number of cells analyzed: control n=109; PP2 n=104; KT5830 n=96; AACoF3 n=103; KT5720 n=87; W7 n=90; calmidazolium n=81; Calmidazolium + W7 n=96 and KN93 n=122. (**b**, **d**) Confocal immunofluorescence images of DDX3X (magenta) in HeLa cells overexpressing TRPV4 and DDX3X and exposed to control conditions or in the presence of GSK and the indicated drugs. Nuclei were stained with DAPI (blue). Colocalization of DDX3X and nuclei is shown in white in the merge panels. (**c**,**e**) Mean nuclear/total DDX3X ratio

obtained under the experimental conditions indicated in HeLa cells overexpressing TRPV4 and DDX3X. The number of cells analyzed for each condition is indicated in each bar. *** $P<0.001$ when comparing isotonic control condition with GSK condition (as determined by two-tailed Student's t-test). Scale bar: 10 μm.

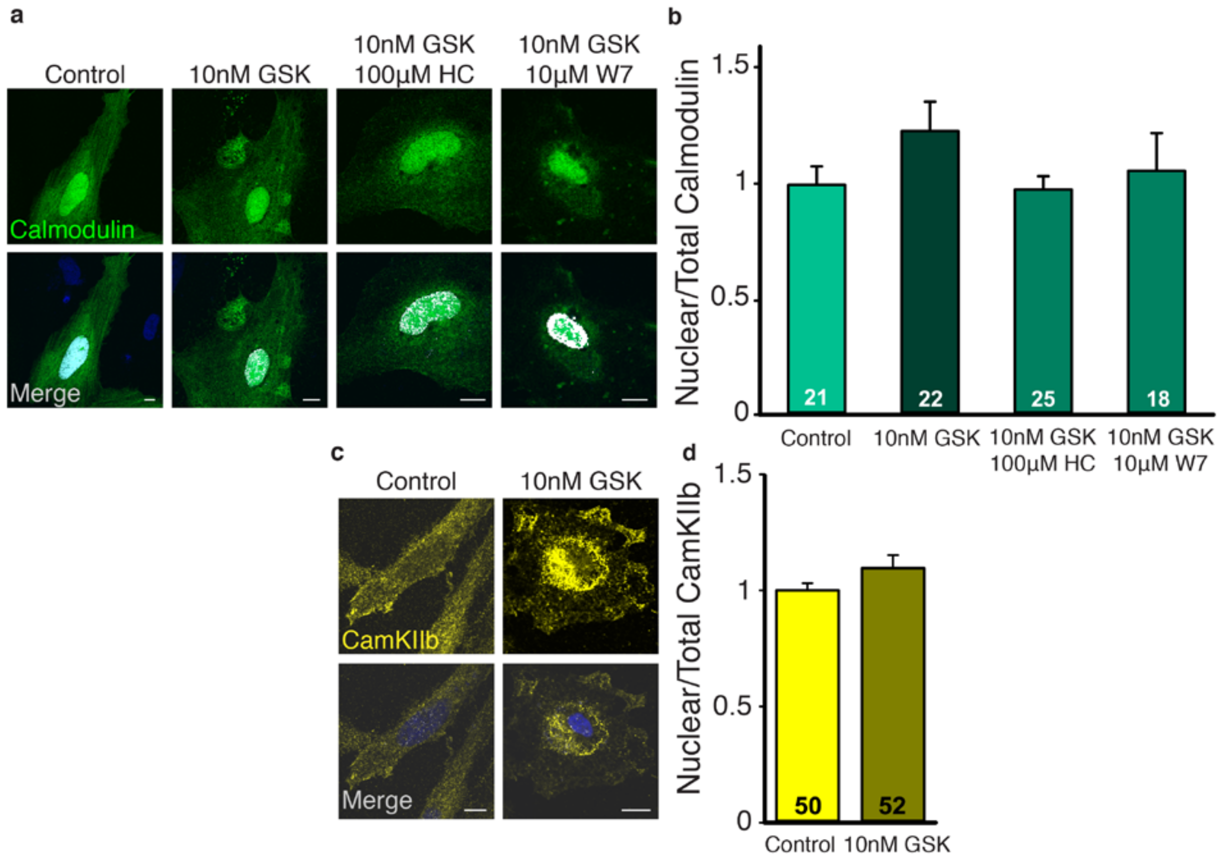

Supplementary Figure 7. Calmodulin and CaMKIIb cellular distribution is insensitive to TRPV4 activation. (a) Confocal immunofluorescence images of calmodulin in HeLa cells overexpressing TRPV4 and DDX3X and exposed to control conditions or in the presence of GSK and the indicated drugs. Nuclei were stained with DAPI (blue). Colocalization of calmodulin and nuclei is shown in white in the merge panels. (**b**) Mean (±SEM) of nuclear/total calmodulin ratio obtained under the experimental conditions indicated in HeLa cells overexpressing TRPV4 and DDX3X. The number of cells analyzed for each condition is indicated in each bar. **(c**) Confocal immunofluorescence images of CamKIIb in HeLa cells overexpressing TRPV4 and DDX3X and exposed to control conditions or in the presence of GSK1016790A. Nuclei were stained with DAPI (blue). Colocalization of CaMKIIb and nuclei is shown in white in the merge panels. (**d**) Mean nuclear/total CaMKIIb ratio obtained under the experimental conditions indicated in HeLa cells overexpressing TRPV4 and DDX3X. The number of cells analyzed for each condition is indicated in each bar. The number of cells analyzed for each condition is indicated in each bar. P>0.05 (not statistically significant) when comparing control condition with any other condition as determined by one way ANOVA followed by a Bonferroni post hoc test (b) or by two-tailed Student's t-test (d). Scale bar: 10 μm.

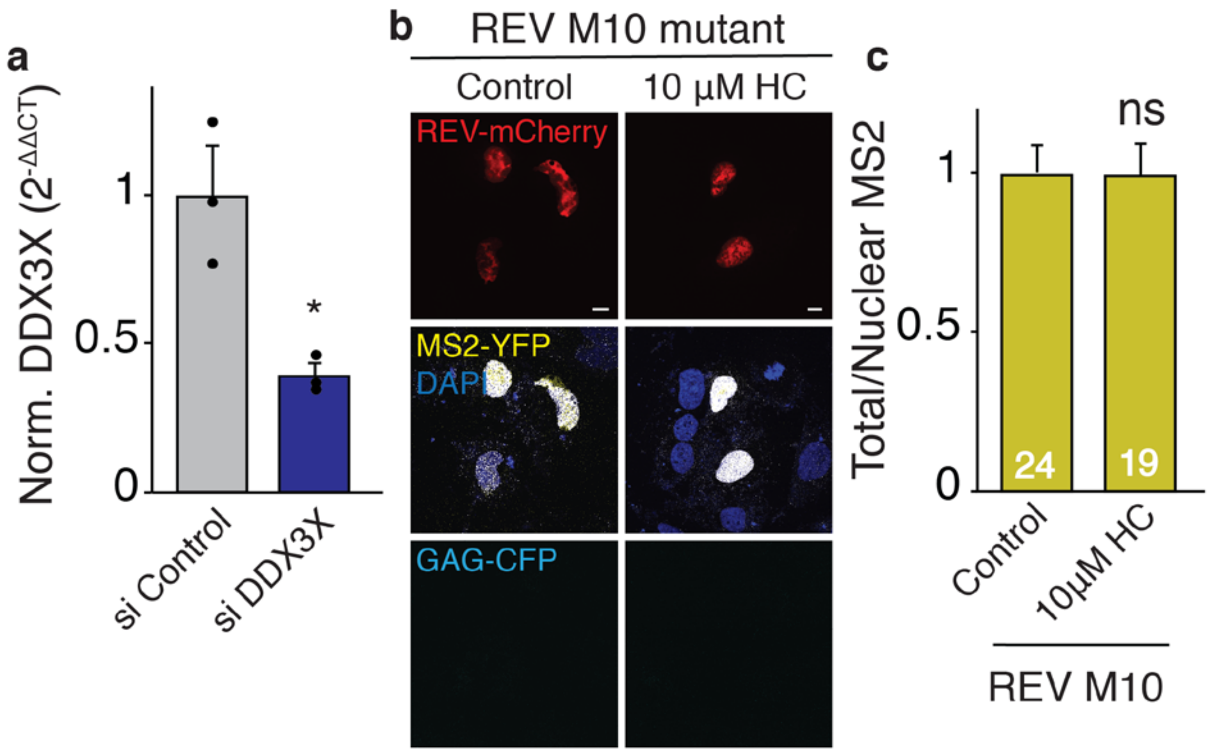

Supplementary Figure 8. Nuclear export and translation of viral RNA. (a) RT-qPCR comparing DDX3X mRNA levels in HeLa cells transfected with siControl and siTRPV4**.** Mean (±SEM) of three independent experiments. **(b)** Representative images of HeLa cells transfected with Rev-mCherry, RRE-gRNA and MS2-YFP, and also stained with DAPI. (**c**) Quantification of nuclear export of gRNA calculated as the ratio of total/nuclear MS2 signal. Data are obtained from 3 independent experiments for each condition with the total number of cells analysed indicated in the bars. * $P<0.05$ when comparing siControl with siDDX3X (a) and control with HC067047 (c) as determined by two-tailed Student's t-test. Scale bar: 10 µm.

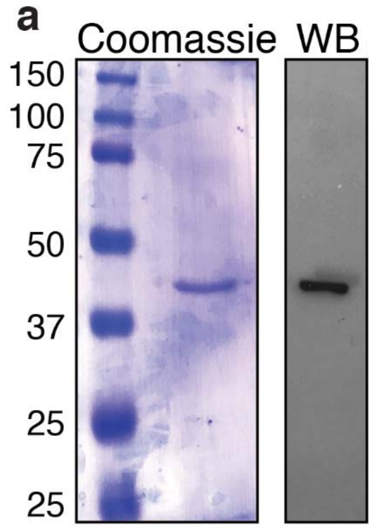

Supplementary Figure 9. Purification of Zika envelop protein. Coomassie staining and western blot using anti-myc antibody to identify the purified Zika envelop protein from cell extracts of HEK293 cells overexpressing Zika envelop protein tagged with Myc.

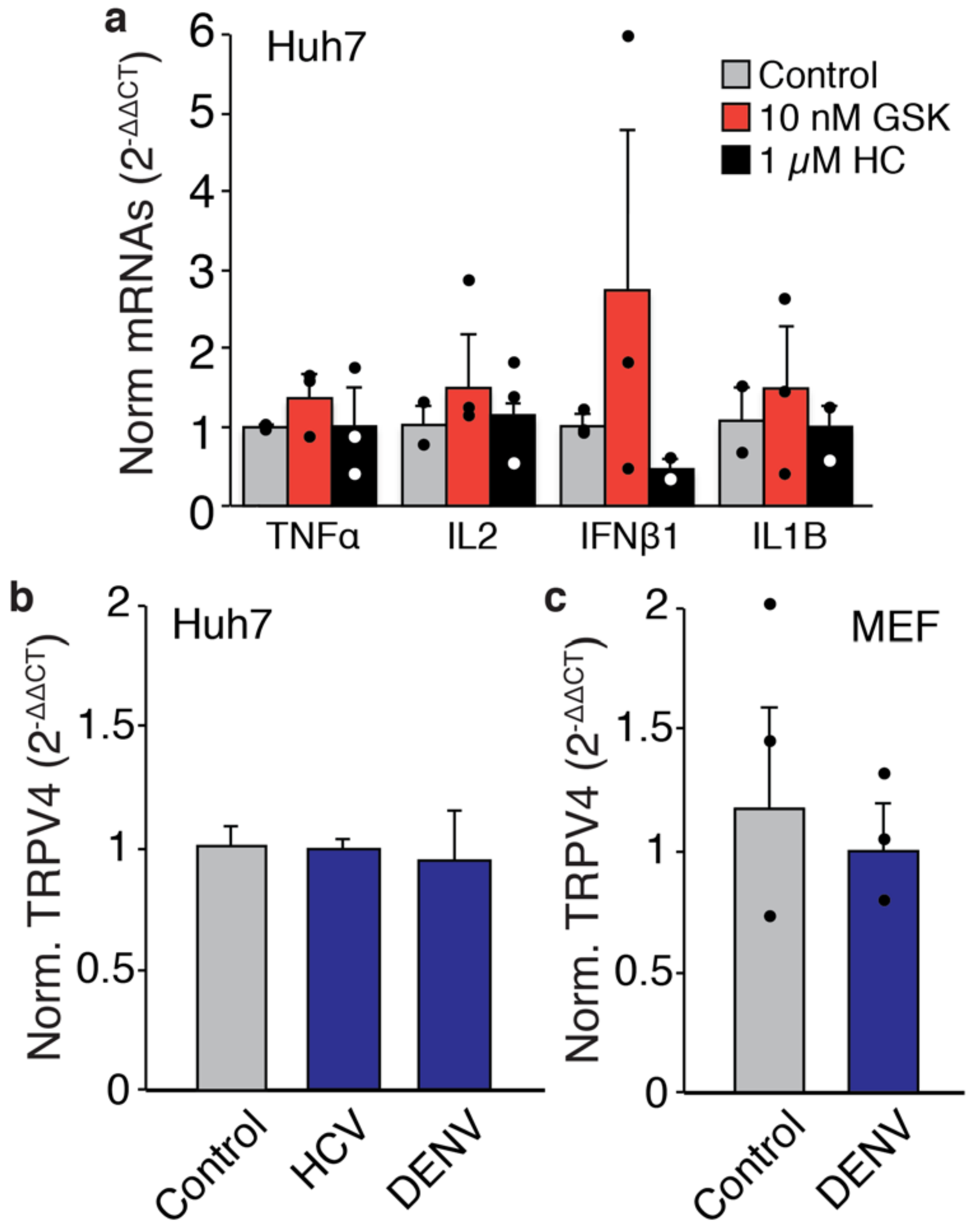

Supplementary Figure 10. Expression of different genes of interest in Huh7 and mouse embryonic fibroblasts. (a) RT qPCR of tumor necrosis factor α, interleukin-2, interleukin 1β and interferon β mRNAs in Huh7 cells exposed to the TRPV4 activator GSK1016790A (10 nM) or the TRPV4 inhibitor HC067047 (10 μM). (**b**) RT-qPCR comparing TRPV4 mRNA levels in Huh7cells infected with HCV or DENV**.** **(c)** RT-qPCR comparing TRPV4 mRNA levels in MEFs infected with DENV**.** Data are Mean (±SEM) obtained from 3 (a, c) and 8 (b) independent experiments for each condition. P>0.2 for all statistical analysis (N.S.), except for the reduction of IFNβ1 in Huh7 cells treated with HC067047 (a) that almost reached statistical significance (P=0.056).

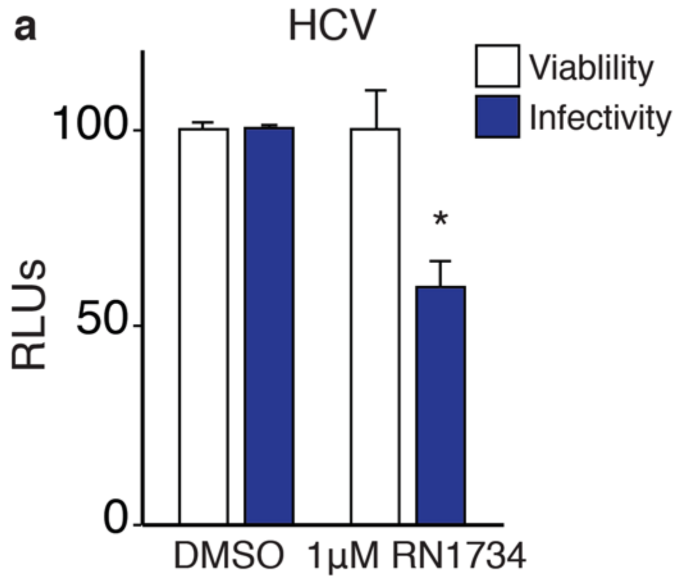

Supplementary Figure 11. DENV infection in Huh7 cells. Cellular viability was measured by CytoTox-Glo cytotoxicity assay and viral infectivity was measured as luciferase activity in Huh7 cells treated with DMSO vehicle or the TRPV4 inhibitor RN1734 (1 μM). Data are Mean (±SEM) of 8 experiments. * $P<0.0001$ when comparing DMSO with RN1734 as determined by two-tailed Student's t-test.

Go-ID	Go-terms	pValue corr	association (% genes)
44419	interspecies interaction between organism	2,38E-03	14
9615	response to virus	3,61E-03	9
51704	multi-organism process	3,61E-03	21
23052	signaling	9,29E-03	49
23033	signaling pathway	9,86E-03	37
60316	positive regulation of ryanodine-sensitive calcium release channel	2,17E-02	2
23034	intracellular signaling pathway	2,19E-02	24
35556	negative regulation of ryanodyne sensitive calcium release channel	3,38E-02	19
60315	lamellipodium assembly	4,21E-02	2
30032	response to other organism	4,21E-02	3
51707	adaptative inmune response based on somatic recombination of inmune receptors	4,21E-02	11
2460	adaptative inmune response	4,21E-02	5
2250	positive regulation of biological process	4,21E-02	5
48518	positive regulation of biological process	4,21E-02	35
10880	regulation of release of sequestered calcium ion into cytosol by sarcoplasmic reticulum	4,21E-02	2
7266	Rho protein signal transduction	4,21E-02	4
7265	Ras protein signal transduction	4,21E-02	6
23046	signaling process	4,21E-02	34
23060	signal transmission	4,21E-02	34
42981	regulation of apoptosis	4,21E-02	18
43065	positive regulation of apoptosis	4,21E-02	12
16310	phosphorylation	4,21E-02	17
43068	positive regulation of programmed cell death	4,21E-02	12
43067	regulation of programed cell death	4,21E-02	18
8154	actin polymerization or depolymerization	4,21E-02	3
10942	positive regulation of cell death	4,21E-02	12
43900	regulation of multi-organism process	4,21E-02	4
30031	cell projection assembly	4,21E-02	5
10941	regulation of cell death	4,21E-02	18
6123	mitochondrial electron transport, cytocrome c to oxigen	4,21E-02	2
6793	phosphorus metabolic process	4,21E-02	19
6796	phosphate metabolic process	4,21E-02	19
51130	positive regulation of cellular compoment organization	4,43E-02	8

Legend

- inmune system response
- signaling
- calcium signaling
- apoptosis
- cellular structures
- metabolism

Supplementary Table 1. List of Go-terms associated with TRPV4 MYTH interactome. We annotated Go-ID reference number, p-value corrected by FDR and the number of genes within the interactome that are associated with that Go-ID. Go-terms were clustered into cellular processes according to the color code described in the legend.

GENE	Forward	Reverse
hACTIN	5'- ACGAGGCCCAGAGCAAGAG-3'	5'-GGTGTGGTGCCAGATTTTCTC-3'
hGADPH	5'-GGAGTCCACTGGCGTCTTC-3'	5'-TGGCTCCCCCCTGAAATG-3'
hHPRT	5'-TGACACTGGCAAAACAATGCA-3'	5'-GGTCCTTTTCACCAGCAAGCT-3'
hTRPV4	5'-CCCGTGAGAACACCAAGTTT-3'	5'-GTGTCCTCATCCGTCACCTC-3'
hDDX3X	5'-TTCTCAGATGTTTGTTGTGTGGATT-3'	5'-AAACTTGCTCAAATGCTATTGCTG-3’
hIL2	5'-AACTCACCAGGATGCTCACA-3'	5'-GCACTTCCTCCAGAGGTTTG-3'
hTNFa	5'-AGCCTCTTCTCCTTCCTGATCGTG-3'	5'-GGCTGATTAGAGAGAGGTCCCTGG-3'
hIFNB1	5'-CTTTCGAAGCCTTTGCTCTG-3'	5'-GGAGAGCAATTTGGAGGAGAC-3'
hIL1B	5'-ACAGATGAAGTGCTCCTTCCA-3'	5'-GTCGGAGATTCGTAGCTGGAT-3'
BMV RNA2	5’-CACGGAACGAGGTTCAATCC-3’	5’-GATAGTAGAAAGAACAAGCACACAACAA-3’
yAct1	5’-TCGTTCCAATTTACGCTGGTT-3’	5’- CGGCCAAATCGATTCTCAA-3’

Supplementary Table 2. Sequences of primers used in the study.
