# Supplementary material for: The TRPV4 channel links calcium influx to DDX3X activity and viral infectivity
Source: Nat Commun. 2018 Jun 13;9:2307. doi: 10.1038/s41467-018-04776-7 (PMC5998047; doi:10.1038/s41467-018-04776-7)
Supplement: Supplementary file 3 — Description of Additional Supplementary Files [file 41467_2018_4776_MOESM3_ESM.pdf]

Description of Additional Supplementary Files:

Supplementary Data 1: Mass spectrometry analysis of DDX3X pulldown assay. The excel file contains under the Classification of pd hit prots worksheet, the proteins identified under control (grey), GSK treated (red) and in black the proteins present in both conditions. The protein worksheet contains all the proteins identified in the assay. The Peptided worksheel contains all the peptides identified in the assay.
